# Supplementary material for: Multibody Model with Foot-Deformation Approach for Estimating Ground Reaction Forces and Moments and Joint Torques during Level Walking through Optical Motion Capture without Optimization Techniques
Source: Sensors (Basel). 2024 Apr 27;24(9):2792. doi: 10.3390/s24092792 (PMC11086093; doi:10.3390/s24092792)
Supplement: Supplementary file 1 [file sensors-24-02792-s001.zip › manuscript_table.pdf]

Table S1. Pearson's correlation coefficients (ρ), root mean square error (RMSE) and relative RMSE (rRMSE) of ground reaction forces (GRFs), moments (GRMs), and joint torques during normal-speed, fast-speed (approximately +20 % of the normal-speed), and slow-speed (approximately -20 % of the normal-speed). The average and standard deviation (SD) for all participants are displayed, with magnitudes normalized to body mass of participants.

Gait speed	Normal			Fast (+20 %)			Slow (-20 %)		
	ρ (SD)	RMSE (SD) [N/kg or N·m/kg]	rRMSE (SD) [%]	ρ (SD)	RMSE (SD) [N/kg or N·m/kg]	rRMSE (SD) [%]	ρ (SD)	RMSE (SD) [N/kg or N·m/kg]	rRMSE (SD) [%]
GRF									
Anterior	0.901 (0.087)	0.427 (0.135)	11.4 (3.9)	0.888 (0.120)	0.551 (0.180)	12.6 (5.0)	0.910 (0.062)	0.356 (0.107)	10.6 (3.4)
Medial	0.813 (0.092)	0.164 (0.029)	17.0 (3.6)	0.776 (0.159)	0.183 (0.034)	17.6 (3.9)	0.748 (0.160)	0.161 (0.027)	18.4 (3.6)
Vertical	0.945 (0.068)	1.422 (0.678)	11.6 (5.4)	0.931 (0.105)	1.614 (0.794)	12.2 (6.0)	0.953 (0.062)	1.258 (0.678)	10.9 (6.2)
GRM									
Frontal	0.796 (0.154)	0.066 (0.028)	19.5 (7.1)	0.753 (0.169)	0.098 (0.056)	23.3 (10.9)	0.790 (0.164)	0.078 (0.049)	21.7 (13.2)
Sagittal	0.906 (0.131)	0.211 (0.080)	13.9 (7.9)	0.910 (0.141)	0.210 (0.088)	12.5 (7.4)	0.856 (0.311)	0.257 (0.336)	14.9 (13.6)
Transverse	0.854 (0.144)	0.018 (0.007)	13.5 (5.4)	0.823 (0.200)	0.023 (0.009)	15.0 (6.4)	0.783 (0.316)	0.020 (0.018)	13.9 (7.9)
Hip									
Frontal	0.897 (0.056)	0.168 (0.046)	14.5 (3.4)	0.866 (0.077)	0.202 (0.065)	15.7 (4.2)	0.914 (0.063)	0.158 (0.042)	13.9 (4.0)
Transverse	0.915 (0.038)	0.032 (0.007)	11.3 (2.6)	0.875 (0.133)	0.046 (0.019)	13.0 (7.1)	0.902 (0.052)	0.032 (0.008)	12.6 (3.2)
Sagittal	0.798 (0.076)	0.307 (0.095)	15.0 (2.5)	0.825 (0.082)	0.416 (0.157)	15.1 (4.1)	0.814 (0.068)	0.219 (0.055)	15.0 (2.7)
Knee									
Frontal	0.878 (0.091)	0.054 (0.023)	16.3 (6.4)	0.839 (0.121)	0.076 (0.047)	19.3 (7.4)	0.876 (0.071)	0.059 (0.021)	18.8 (5.7)
Transverse	0.860 (0.120)	0.040 (0.016)	18.4 (7.0)	0.817 (0.209)	0.060 (0.048)	20.5 (9.4)	0.870 (0.123)	0.040 (0.019)	18.6 (7.2)
Sagittal	0.759 (0.084)	0.187 (0.053)	18.3 (3.7)	0.802 (0.083)	0.215 (0.047)	16.3 (3.5)	0.760 (0.148)	0.160 (0.044)	17.9 (5.9)
Ankle									
Frontal	0.750 (0.245)	0.039 (0.016)	22.1 (7.2)	0.725 (0.214)	0.057 (0.040)	24.0 (9.3)	0.809 (0.121)	0.042 (0.018)	23.0 (7.1)
Transverse	0.855 (0.135)	0.053 (0.024)	17.9 (6.9)	0.814 (0.206)	0.078 (0.056)	21.0 (10.4)	0.873 (0.122)	0.056 (0.025)	18.5 (7.4)
Sagittal	0.938 (0.123)	0.186 (0.096)	10.8 (8.5)	0.941 (0.137)	0.177 (0.115)	9.5 (9.3)	0.941 (0.104)	0.170 (0.090)	10.4 (8.8)
